# Geriatric Patient Successfully Managed With Conservative Therapy Despite Portal Venous Gas

**DOI:** 10.7759/cureus.110043

**Published:** 2026-06-01

**Authors:** Marta S Stega, Ehssan Zare

**Affiliations:** 1 General Surgery, Henry Ford Providence, Southfield, USA; 2 General Surgery, Trauma Surgery, Critical Care, Henry Ford Providence, Southfield, USA

**Keywords:** conservative management, emphysematous gastritis, gastric emphysema, gastric pneumatosis, portal venous gas

## Abstract

Intramural gastric air with concomitant portal venous gas is an uncommon radiographic finding that presents a significant diagnostic challenge. The primary differential diagnoses include gastric emphysema (GE), a generally benign and self-limiting condition, and emphysematous gastritis (EG), a rare but potentially fatal infectious process associated with high mortality rates. Although these conditions may appear similar on computed tomography (CT), they differ considerably in pathophysiology, clinical presentation, management, and prognosis. Accurate distinction between the two entities is essential to guide treatment decisions and avoid unnecessary surgical intervention.

We present the case of a 96-year-old man with a history of chronic heart failure and hypertension who presented with a 24-hour history of nausea, vomiting, abdominal pain, and diarrhea following a meal. On admission, the patient was hemodynamically stable, afebrile, and without signs of peritonitis. Laboratory evaluation was notable only for acute kidney injury secondary to dehydration. CT imaging of the abdomen and pelvis demonstrated gastric distention, intramural gastric air, and significant portal venous gas, raising concern for both GE and EG.

Despite concerning radiographic findings, the patient’s benign clinical presentation favored gastric emphysema. He was managed conservatively with bowel rest, intravenous fluids, proton pump inhibitor therapy, broad-spectrum antibiotics, serial abdominal examinations, and close clinical observation. Throughout hospitalization, the patient remained clinically stable with progressive improvement in symptoms and no evidence of systemic toxicity or acute abdomen. Repeat CT imaging obtained on hospital day 3 demonstrated complete resolution of portal venous gas and near-complete resolution of gastric wall air. The patient tolerated diet advancement and was discharged home in stable condition.

This case emphasizes the importance of correlating radiographic findings with the overall clinical presentation when evaluating gastric pneumatosis and portal venous gas. Careful clinical assessment and close monitoring may allow successful conservative management in appropriately selected patients while avoiding unnecessary operative intervention.

## Introduction

The presence of intramural gastric air and portal venous gas is an uncommon radiographic finding that presents a significant diagnostic and therapeutic challenge. The two principal diagnostic considerations are gastric emphysema (GE) and emphysematous gastritis (EG). Although these entities may demonstrate similar imaging characteristics on computed tomography (CT), they differ considerably in underlying pathophysiology, clinical severity, management strategies, and overall prognosis. Consequently, accurate and timely differentiation between the two conditions is essential for appropriate patient care [1-4].

GE is generally considered a benign and self-limiting process resulting from the dissection of air into the gastric wall, often secondary to mucosal disruption or increased intraluminal pressure. In contrast, EG represents a rare but potentially life-threatening infectious process caused by the invasion of the gastric wall by gas-forming microorganisms. Mortality rates associated with EG remain high despite advances in imaging, critical care, and antimicrobial therapy [1,3].

Because radiographic findings alone may not reliably distinguish between these two conditions, clinical correlation is of critical importance. Factors such as hemodynamic stability, abdominal examination findings, laboratory abnormalities, and progression of symptoms often play a decisive role in guiding diagnosis and management [2-4]. This report describes a rare case of GE associated with portal venous gas in an elderly patient who was successfully managed conservatively despite concerning imaging findings suggestive of EG. 

## Case presentation

A 96-year-old male with a past medical history significant for chronic heart failure and hypertension presented to the emergency department with a 24-hour history of nausea, vomiting, abdominal pain, and non-bloody diarrhea. The patient reported that the onset of symptoms occurred shortly after consumption of a meal. The vomiting was nonbloody and nonbilious, and there was no history of hematemesis, melena, recent instrumentation, alcohol abuse, or ingestion of corrosive substances. 

The patient’s home medications included aspirin 81 mg daily, atenolol 50 mg daily, furosemide 40 mg daily, terazosin 10 mg daily, atorvastatin 20 mg daily, and carvedilol 3.125 mg twice daily. The patient denied taking any additional medications and reported adherence to the prescribed medication regimen.

Upon presentation, the patient appeared clinically stable and in no acute distress. Vital signs demonstrated a blood pressure of 105/72 mmHg, heart rate of 81 beats per minute, oxygen saturation of 100% on room air, and temperature of 97.2 °F. Physical examination of the abdomen revealed mild, diffuse, colicky tenderness localized predominantly to the periumbilical region. The abdomen was soft and nondistended, without guarding, rebound tenderness, or other signs suggestive of peritonitis. Bowel sounds were preserved.

Initial diagnostic evaluation included comprehensive laboratory studies. Serum creatinine was elevated at 2.5 mg/dL, initially presumed to be due to acute kidney injury secondary to dehydration from gastrointestinal fluid losses (Table 1).

**Table 1:** Comprehensive laboratory results at the time of admission, with reference ranges Alk Phos: alkaline phosphate; AST: aspartate aminotransferase; ALT: alanine aminotransferase

Test	Result	Unit	Normal Range
WBC	9.00	K/mcL	4.0–11.0 K/mcL
HbG	12.5	gm/dL	12.0–16.0 gm/dL
Platelet	131	K/mcL	150–450 K/mcL
BUN	35	mg/dL	7–20 mg/dL
Creatinine	2.5	mg/dL	0.6–1.3 mg/dL
Sodium level	135	mmol/L	135–145 mmol/L
Potassium level	4.8	mmol/L	3.5–5.0 mmol/L
Calcium level	10.6	mg/dL	8.5–10.5 mg/dL
Alk Phos	81	unit/L	44–147 unit/L
AST	65	unit/L	10–40 unit/L
ALT	38	unit/L	7–56 unit/L
Total protein	9.1	gm/dL	6.0–8.3 gm/dL
Lactic acid	2.2	mmol/L	0.5–2.2 mmol/L

**Figure 1:**
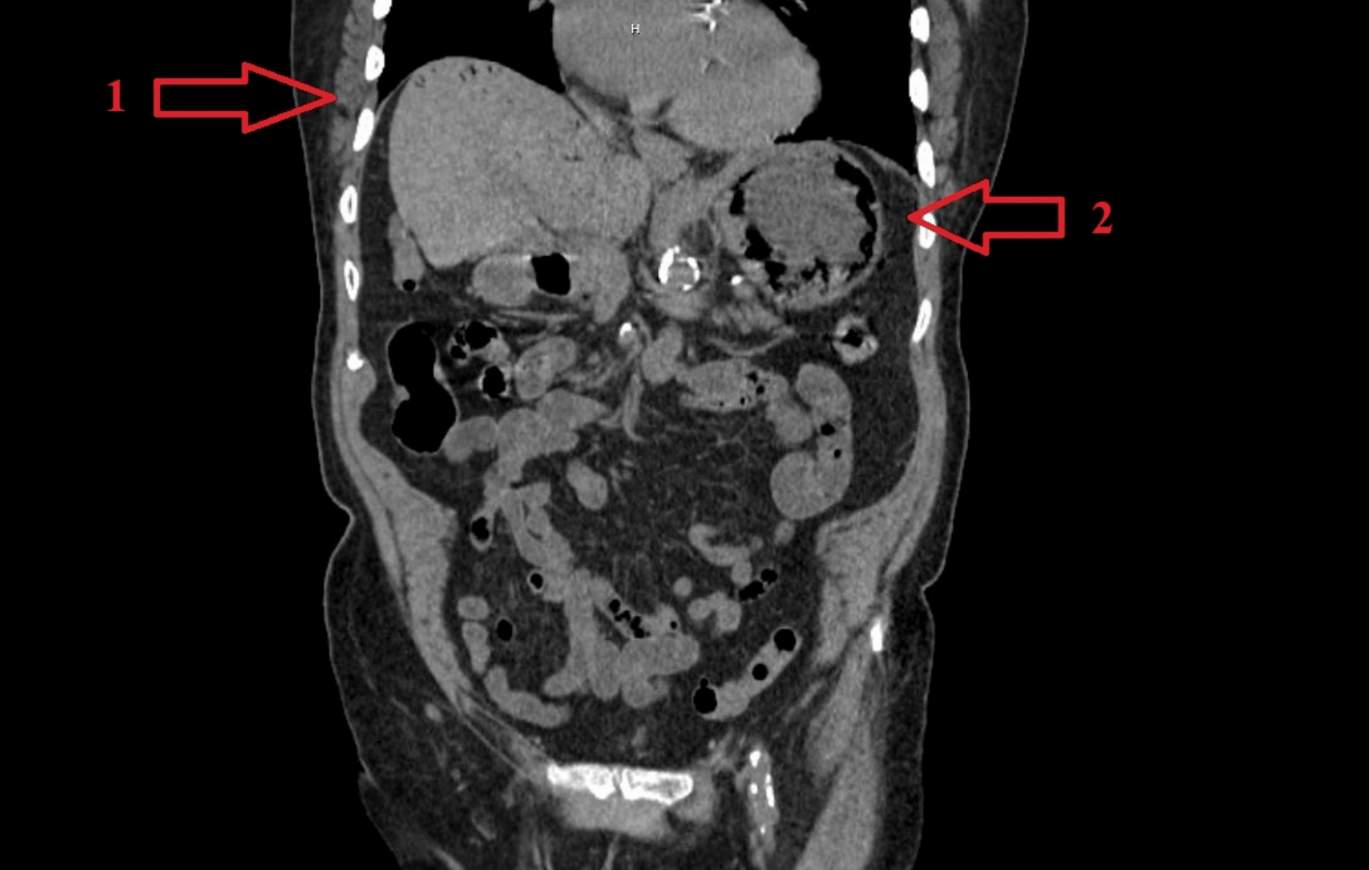
Air within the gastric wall and portal venous gas (coronal view) Arrow 1 points at portal venous gas. Arrow 2 points at intramural gastric air.

**Figure 2:**
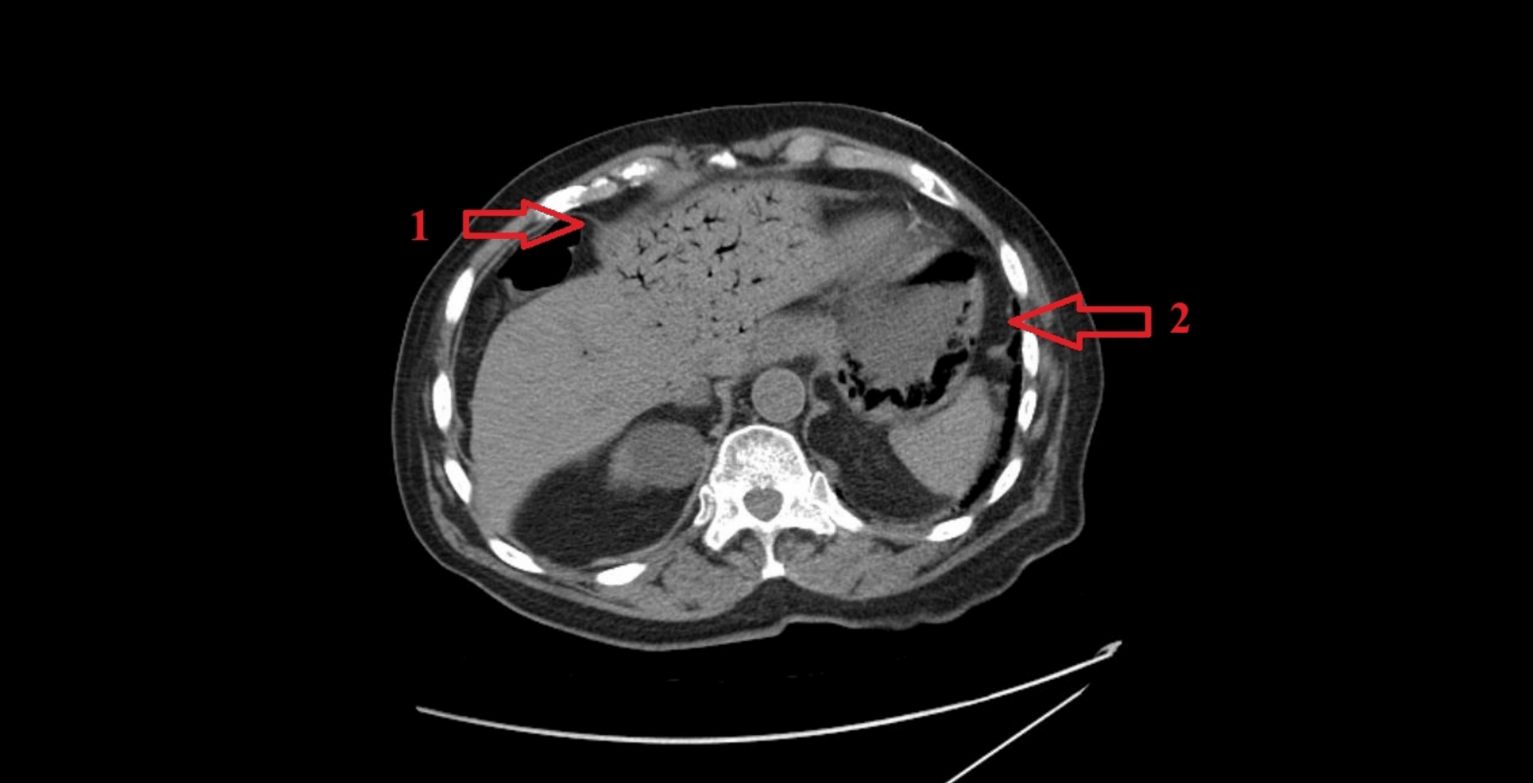
Portal venous gas and air within the gastric wall (axial view) Arrow 1 points at portal venous gas. Arrow 2 points at intramural gastric air.

Computed tomography (CT) of the abdomen and pelvis demonstrated marked gastric distention with extensive air within the gastric wall as well as significant portal venous gas involving the hepatic portal venous system (Figures 1, 2). The imaging findings raised concern for either GE or EG.

The patient was admitted for close clinical observation and initially managed conservatively with nil per os (NPO) status, intravenous fluid resuscitation, and proton pump inhibitor therapy. Empiric broad-spectrum intravenous antibiotics were initiated due to the inability to definitively rule out EG. Antibiotics consisted of ceftriaxone 2000 mg every 24 hours and metronidazole 500 mg every 8 hours. At the time of admission, the patient’s nausea and vomiting had resolved; therefore, nasogastric decompression was deferred, although a low threshold for placement was maintained should his clinical condition worsen. 

Given the potentially life-threatening nature of EG, serial abdominal examinations, repeated laboratory assessments, and continuous monitoring of hemodynamic status were performed over the subsequent 48 hours. Particular attention was directed toward identifying signs of clinical deterioration, including worsening abdominal pain, fever, metabolic acidosis, or the development of peritonitis.

Throughout hospitalization, the patient remained hemodynamically stable and afebrile. He experienced no recurrence of nausea or vomiting, and his abdominal pain progressively improved. Serial abdominal examinations consistently demonstrated a soft, nondistended abdomen without guarding or rebound tenderness. The patient also maintained normal bowel function (continued to report no blood in the bowel movements) during his hospital course. Repeat laboratory studies obtained following fluid resuscitation are summarized in Table 2. The patient’s creatinine level improved from 2.5 to 1.4, supporting the diagnosis of acute kidney injury secondary to dehydration. However, the persistent mild elevation in creatinine was presumed to reflect underlying, previously undiagnosed, chronic kidney disease. 

**Table 2:** Comprehensive laboratory results on the day of discharge from the hospital, with reference ranges Lactic acid results from the day after admission; no further trend of the result was acquired due to the normal value. Provided Alk Phos, AST, ALT, and total protein results are from admission day 2, as they were not trended further. Alk Phos: alkaline phosphate; AST: aspartate aminotransferase; ALT: alanine aminotransferase

Test	Result	Unit	Normal Range
WBC	11.3	K/mcL	4.0–11.0 K/mcL
HbG	12.5	gm/dL	12.0–16.0 gm/dL
Platelet	143	K/mcL	150–450 K/mcL
BUN	17	mg/dL	7–20 mg/dL
Creatinine	1.4	mg/dL	0.6–1.3 mg/dL
Sodium level	136	mmol/L	135–145 mmol/L
Potassium level	4	mmol/L	3.5–5.0 mmol/L
Calcium level	8.5	mg/dL	8.5–10.5 mg/dL
Alk Phos	51	unit/L	44–147 unit/L
AST	49	unit/L	10–40 unit/L
ALT	21	unit/L	7–56 unit/L
Total protein	5.7	gm/dL	6.0–8.3 gm/dL
Lactic Acid	1.3	mmol/L	0.5–2.2 mmol/L

**Figure 3:**
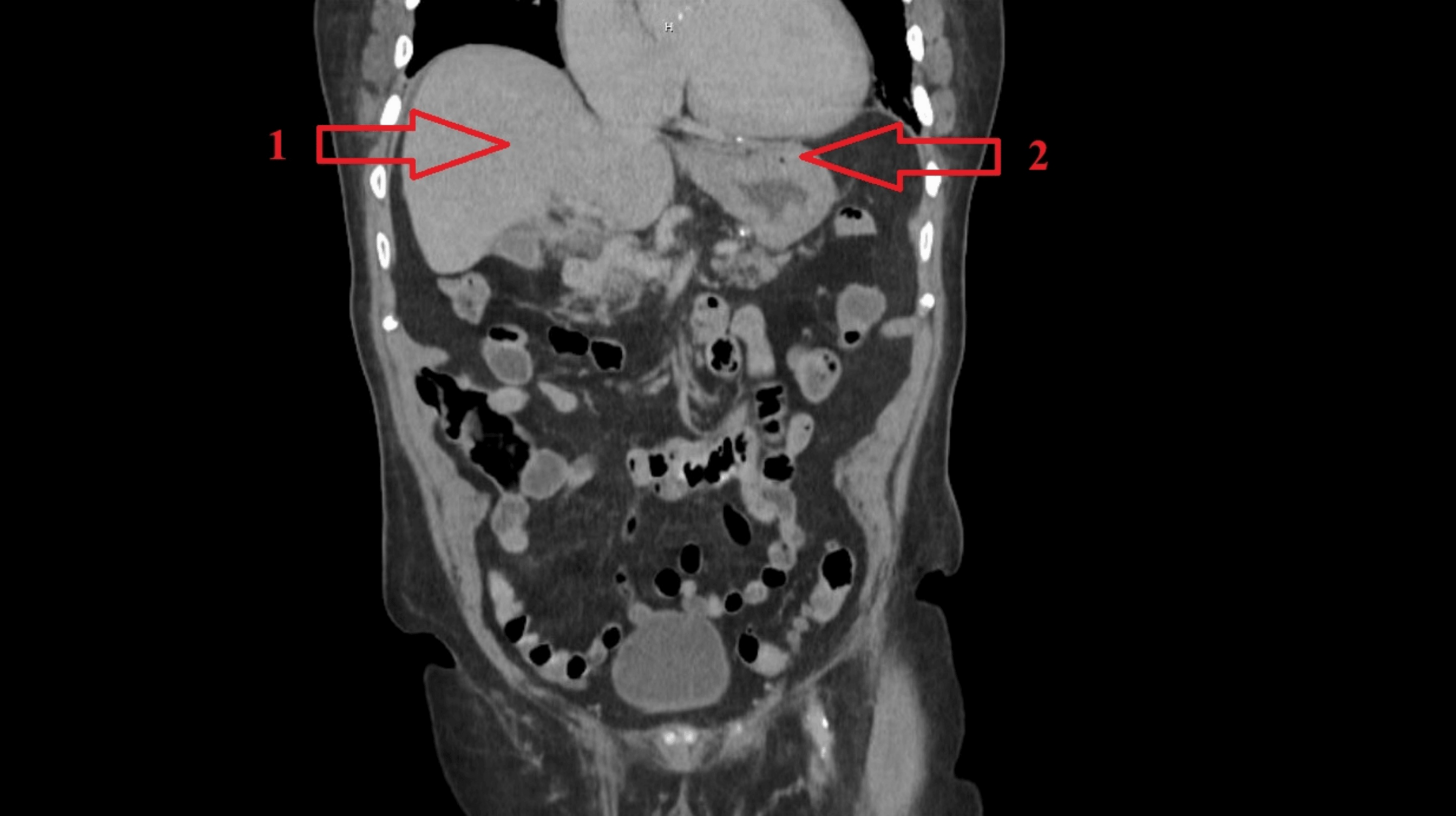
Resolution of portal venous gas (arrow 1) and residual air within the gastric wall (arrow 2): coronal view

**Figure 4:**
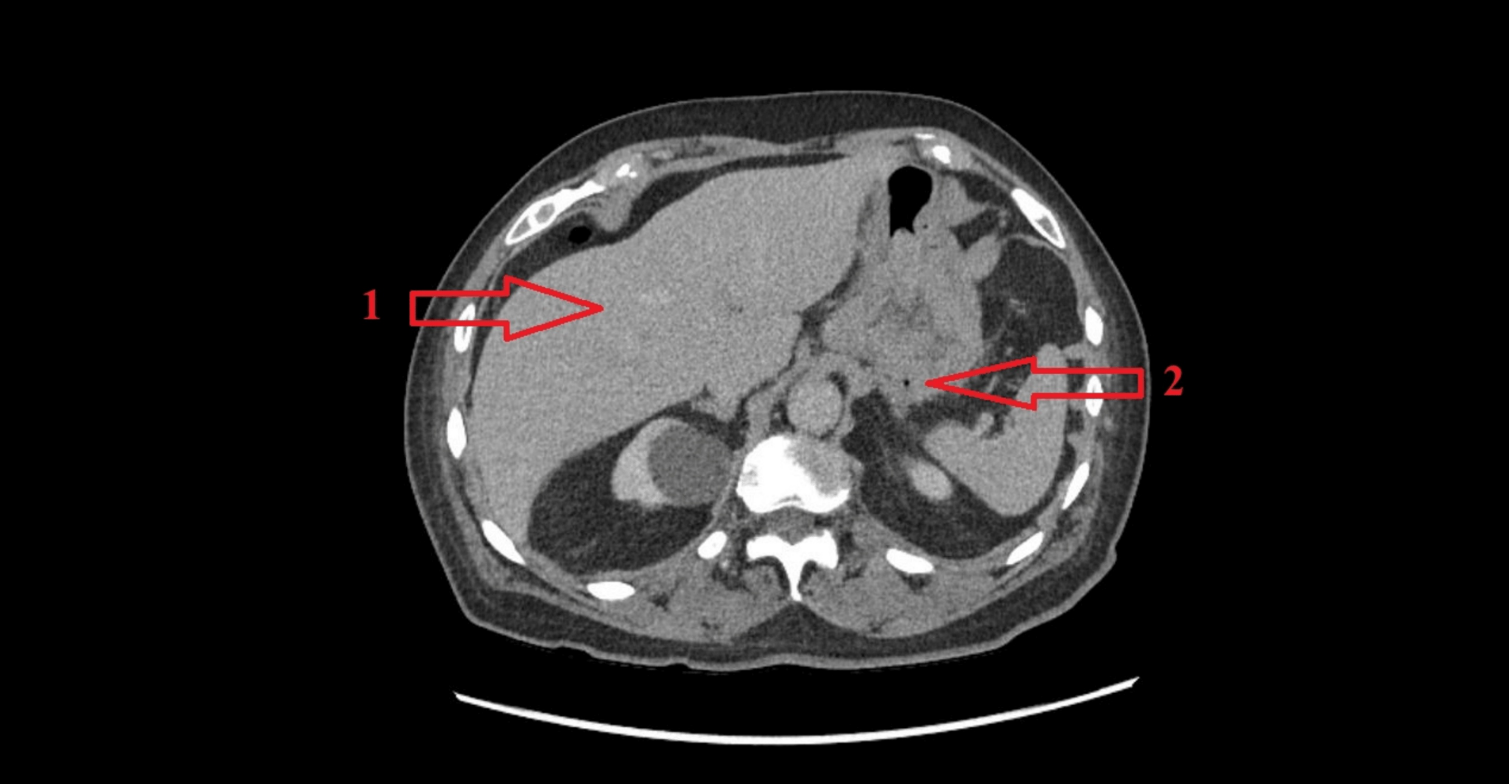
Resolution of a portal venous gas (arrow 1) and residual air within the gastric wall (arrow 2): axial view

Repeat CT imaging obtained on admission day 3 demonstrated complete resolution of the portal venous gas and significant interval improvement in the intramural gastric air (Figures 3, 4). Given the marked radiographic and clinical improvement, the patient was gradually advanced to a clear liquid diet, which he tolerated without difficulty. His diet was subsequently advanced to a gastrointestinal soft diet.

Following continued clinical stability and tolerance of oral intake, the patient was discharged home with family support and on empiric oral antibiotics (one-week course of oral amoxicillin-clavulanate (875 mg/125 mg)) prescribed by the primary physician. No clear clinical rationale for this management approach was documented beyond alignment with the physician’s usual practice pattern. 

In light of the benign clinical course, absence of systemic toxicity, and rapid radiographic improvement with conservative therapy, the final diagnosis was determined to be GE without a definite, identifiable underlying etiology.

## Discussion

EG is a rare but severe form of gastritis characterized by gas formation within the gastric wall secondary to invasion by gas-producing microorganisms. First described by Fraenkel in 1889, EG remains associated with significant morbidity and mortality, with reported mortality rates ranging from 55% to 61%. Predisposing risk factors include diabetes mellitus, chronic kidney disease, immunosuppression, alcohol abuse, recent abdominal surgery, long-term corticosteroid therapy, nonsteroidal anti-inflammatory drug use, and ingestion of corrosive substances [1]. In our patient, previously undiagnosed chronic kidney disease was identified; however, no additional risk factors were identified. 

The most commonly implicated organisms include *Streptococcus spp., Escherichia coli, Enterobacter spp., Clostridium spp., Pseudomonas aeruginosa, Staphylococcus aureus, Sarcina spp., Candida spp., *and *Mucor spp* [2]. These organisms invade the gastric mucosa, resulting in inflammation, tissue ischemia, and intramural gas production. Patients with EG typically present with severe abdominal pain, nausea, vomiting, abdominal distention, fever, leukocytosis, and systemic signs of toxicity. 

Computed tomography remains the diagnostic imaging modality of choice because of its sensitivity in identifying intramural gas and associated intra-abdominal pathology. Radiographic findings suggestive of EG include diffuse gastric wall thickening, irregular or mottled intramural gas patterns, and associated intestinal pneumatosis [3]. Portal venous gas is also more commonly associated with EG and may indicate severe gastrointestinal pathology.

GE, in contrast, is generally a noninfectious process caused by the dissection of air into the gastric wall. GE may occur secondary to increased intragastric pressure, mucosal injury, obstruction, trauma, or pulmonary air dissection. GE has traditionally been categorized into obstructive, traumatic, and pulmonary subtypes [4]. Obstructive causes include gastric outlet obstruction, volvulus, malignancy, and strictures. Traumatic causes are commonly related to endoscopic procedures, nasogastric tube placement, or blunt abdominal trauma. Pulmonary-type GE occurs when air from ruptured alveoli tracks along vascular planes into the mediastinum and subsequently into the gastric wall [4]. Forceful vomiting was also described in the literature as a possible reason for mucosal tears and increased intragastric pressure that can lead to GE [5]. We were unable to definitively establish the underlying etiology of the gastric wall gas or portal venous gas in our patient. However, the patient did report multiple episodes of vomiting prior to admission, which may have served as the inciting factor through increased intragastric pressure. 

The presence of portal venous gas can be found in association with both GE and EG [1-6]. It is believed, however, that it arises based on different mechanisms. In GE, the proposed mechanism is believed to be mechanical rather than infectious in nature [2]. Increased intragastric pressure, such as that caused by forceful vomiting, can result in disruption of the gastric mucosal barrier. This allows intraluminal air to dissect into the gastric wall and subsequently enter the venous circulation, ultimately tracking into the portal venous system [4]. In contrast, portal venous gas associated with EG is thought to result from gas production by invasive organisms described above in the setting of gastric wall infection and inflammation. 

Although hepatic portal venous gas has traditionally been associated with life-threatening pathologies, such as bowel ischemia, emerging literature suggests that conservative management may be appropriate in carefully selected, clinically stable patients [2,4,6]. Current evidence further indicates that the severity of radiographic findings alone should not necessarily dictate the need for operative intervention [4].

Although EG and GE may initially appear radiographically similar, several imaging characteristics may assist in differentiation. GE classically presents as linear or curvilinear collections of air within the gastric wall without significant wall thickening. Conversely, EG more commonly demonstrates irregular, mottled, or bubbly intramural gas associated with inflammatory gastric wall thickening and systemic manifestations of infection [2]. Nevertheless, radiographic findings must always be interpreted in conjunction with the patient’s clinical condition. 

## Conclusions

In the present case, the linear appearance of intramural gastric air favored gastric emphysema; however, the concomitant portal venous gas initially raised concern for emphysematous gastritis. Despite these alarming imaging findings, the patient’s benign abdominal examination, stable hemodynamic status, absence of systemic toxicity, and rapid clinical improvement strongly supported conservative management.

This case underscores the importance of integrating radiographic findings with the overall clinical presentation when evaluating patients with gastric pneumatosis and portal venous gas. Although portal venous gas is often associated with bowel ischemia and severe intra-abdominal pathology, it may also occur in relatively benign conditions. Careful clinical assessment and risk stratification of each patient, serial abdominal examinations, close hemodynamic monitoring, and interval imaging can help identify patients who may be safely managed nonoperatively while avoiding unnecessary surgical intervention. In our patient’s case, if surgical intervention had become necessary, a goals-of-care discussion would have been essential. Given the patient’s advanced age and multiple comorbidities, he would have represented a very high-risk surgical candidate.
